# Chronic Nitrogen Deposition Has a Minor Effect on the Quantity and Quality of Aboveground Litter in a Boreal Forest

**DOI:** 10.1371/journal.pone.0162086

**Published:** 2016-08-31

**Authors:** Nadia I. Maaroufi, Annika Nordin, Kristin Palmqvist, Michael J. Gundale

**Affiliations:** 1 Department of Forest Ecology and Management, Swedish University of Agricultural Sciences (SLU), Umeå, Sweden; 2 Umeå Plant Science Center (UPSC), Department of Forest Genetics and Plant Physiology, Swedish University of Agricultural Sciences, Umeå, Sweden; 3 Department of Ecology and Environmental Science (EMG), Umeå University, Umeå, Sweden; University of Illinois at Chicago, UNITED STATES

## Abstract

There is evidence that anthropogenic nitrogen (N) deposition enhances carbon (C) sequestration in boreal soils. However, key underlying mechanisms explaining this increase have not been resolved. Two potentially important mechanisms are that aboveground litter production increases, or that litter quality changes in response to N enrichment. As such, our aim was to quantify whether simulated chronic N deposition caused changes in aboveground litter production or quality in a boreal forest. We conducted a long-term (17 years) stand-scale (0.1 ha) forest experiment, consisting of three N addition levels (0, 12.5, and 50 kg N ha^-1^ yr^-1^) in northern Sweden, where background N deposition rates are very low. We measured the annual quantity of litter produced for 8 different litter categories, as well as their concentrations of C, N, phosphorus (P), lignin, cellulose and hemi-cellulose. Our results indicate that mosses were the only major litter component showing significant quantitative and qualitative alterations in response to the N additions, indicative of their ability to intercept a substantial portion of the N added. These effects were, however, offset by the other litter fractions where we found no changes in the total litter fluxes, or individual chemical constituents when all litter categories were summed. This study indicates that the current annual litter fluxes cannot explain the increase in soil C that has occurred in our study system in response to simulated chronic N application. These results suggest that other mechanisms are likely to explain the increased soil C accumulation rate we have observed, such as changes in soil microbial activity, or potentially transient changes in aboveground litter inputs that were no longer present at the time of our study.

## Introduction

Combustion of fossil fuels, production of fertilizers, and agricultural intensification have substantially increased the global emissions of reactive nitrogen (N_r_) released into the atmosphere in the forms of NH_x_ and NO_y_ [1,2]. A portion of these N_r_ emissions eventually enter the biosphere through deposition, and there is an increasing interest in understanding how anthropogenic N_r_ deposition alters the global carbon (C) and nitrogen (N) cycles [2–6]. Nitrogen is one of the most limiting nutrients for primary production in many terrestrial environments such as boreal and temperate forests, due to slow soil mineralization and biological N_2_ fixation rates [7–9]. The increase of anthropogenic N_r_ deposition has been proposed to alleviate N limitation, and thus enhance tree productivity and aboveground C storage in these ecosystems [10,11]. Results from long-term N addition studies indicate that in tropical, temperate and boreal forests, the quantity of C sequestered in standing vegetation per unit of N deposition ranges from 5–30 kg C kg N^-1^ [11–16]. In addition to enhanced C storage in standing biomass, a few long-term N addition studies have also shown that sequestration of C into soils can increase in the range of 10–25 kg^-1^ C kg N^-1^ in boreal, temperate and tropical forests [13,14,17,18]. This finding has significant implications for the global C cycle, because boreal soils represent a larger and more stable C pool than boreal vegetation (3–6 fold greater C pool size) [19,20].

While there is increasing evidence that anthropogenic N_r_ deposition enhances C sequestration in forest soils, key mechanisms driving this increase have not been resolved [21]. One specific mechanism by which N addition has been proposed to enhance soil C sequestration is by increasing plant growth, thereby potentially increasing aboveground litter inputs to soil [21,22]. Nitrogen fertilization has been shown to increase canopy leaf area and reduce needle life span in coniferous forests [23–26]. In addition to canopy litter, understory vegetation such as ericaceous dwarf-shrubs (e.g. *Vaccinium myrtillus*) and bryophytes are also important sources of litter in boreal forests, and these inputs may also change in response to N [27,28], through shifts in plant species composition or biomass [29]. For instance, Gundale et al. [30] and Palmroth et al. [31] showed that N enrichment caused a decrease of *V*. *myrtillus* and bryophyte biomasses in response to N, likely resulting in reduced understory litter inputs that might offset increases of canopy litter. A meta-analysis by Janssens et al. [21] showed that high dose (37–290 kg N ha^-1^ yr^-1^) N fertilization experiments have highly variable effects on aboveground litter inputs, suggesting that substantial uncertainty remains regarding the impact of N deposition on aboveground litter production.

In addition to changing litter production rates, N enrichment could also alter the quality of litter entering the soil, which in turn could influence litter decomposition rates, and soil C accumulation and stability [32]. In boreal forests, N fertilization typically increases foliar N concentrations, as well as concentrations of other elements [33]. For example, elemental ratios such as C:N are well known to control litter decomposition rates [34,35]. Enhanced N acquisition by plants may also lead to increases in N:P ratios, which may stimulate phosphorus (P) uptake and turnover through litter, as plants attempt to balance their stoichiometry [36,37]. Further, lignin and its ratio with N or cellulose have been shown to be good predictors of litter decomposition rates [35,38–40], and some studies have shown that N can cause a reduction in lignin concentrations [41,42], thereby potentially accelerating decomposition and reducing soil C accumulation rates through mineralization and respiration processes [40,43]. In contrast, more recent studies argued that these shifts in litter stoichiometry may instead enhance soil C accumulation, through increased degradation efficacy of microbes [44,45]. Microbes may increase the proportion of litter products being processed and incorporated into their microbial biomass, which potentially accumulate and further stabilized within the mineral soil layer [44,46]. These studies demonstrate that uptake of anthropogenic N by trees and understory vegetation may not only impact the quantity of litter produced, but also its chemical quality, both of which have been poorly investigated within experimental settings using chronic low-dose N experiments designed to simulate atmospheric N deposition.

In this study, we used a long-term (17-year) replicated N addition experiment maintained since 1996 in northern Sweden to assess how atmospheric N_r_ deposition affects both the quantity and quality of annual litter production in a northern boreal forest. We established control plots and two N addition treatments consisting of 12.5 and 50 kg N ha^-1^ yr^-1^ (low and high N treatments, respectively) [47,48], with the low N treatment representing upper level N_r_ deposition rates currently observed in the boreal region [49,50], and the high N treatment level serving as a useful comparison with many previous long-term forest fertilization experiments in the boreal region (e.g. [11,12]). We tested the following hypotheses: 1) That chronic N addition will cause litter quality to change, such that N concentration will increase in all litters, and lignin, C:N, lignin:N, lignin:hemi-cellulose/cellulose ratios will decrease. Further, we expect an increase of P concentration in litter tissues, and reduction in C:P and lignin:P ratios. 2) That long-term N addition will have a positive effect on annual quantity of litter produced by trees; whereas, we anticipate a negative effect on bryophyte and shrub litter production, particularly in the high N treatment, because previous work from this study system has revealed lower abundances of these species in high N plots than in control plots [16,30]. 3) That total annual soil input of C, N, P, lignin, cellulose and hemi-cellulose via all types of aboveground litter combined (i.e. quantity x quality) will increase in response to N addition, with a greater increase occurring in response to the high N addition relative to the low N addition treatment. We expect increases to occur for all litter chemical variables (even lignin), because we expect that increases in litter quantity will be more pronounced than any changes in litter quality that may occur. By testing these three hypotheses, we explicitly address one potential mechanism that may help explain the significant increase in soil C induced by the N treatments that we previously described in this study system [18].

## Materials and Methods

### Site characteristics

The study was carried out at Svartberget Experimental Forest (64°14´ N, 19°46´E) in the middle boreal zone of northern Sweden [51]. No specific permission was required, and the field study did not involve endangered or protected species. The background atmospheric N_r_ deposition in this region is approximately 2 kg N ha^-1^ yr^-1^ [52] and the mean annual precipitation for the experimental site is 583 mm. The forest is a late successional (~120 years old) Norway spruce forest (*Picea abies* (L.) Karst) with occasional Scots pine (*Pinus sylvestris* L.), Birch (*Betula pendula* Roth.) and Aspen (*Populus tremula* L.) throughout. The understory layer is dominated by ericaceous species, *Vaccinium myrtillus* L. and to a lesser extent by *V*. *vitis-idaea* L. and the grass species, *Deschampsia flexuosa* (L.) Trin [16]. The moss-mat consists primarily of *Hylocomium splendens* (Hedw.) B.S.G, *Pleurozium schreberi* (Bird), and *Ptilium crista-castrensis* (Hedw.). Soils are podzols formed from glacial till [53].

### Experimental design

In 1996, we established a randomized block design experiment, consisting of six blocks, with each containing three 31.6×31.6 m plots (0.1 ha) randomly assigned a N addition treatment (0, 12.5, or 50 kg N ha^-1^ yr^-1^; as control, low, and high, respectively) [47,54]. Each 0.1 ha plot had a surrounding buffer zone of ≥32 m. For the current experiment, we excluded *a priori* one of the six blocks because it exhibited significantly different soil characteristics (i.e. poorly drained soil) [18]. The long-term N addition treatments have been manually applied every year since 1996 by spreading solid granules of ammonium nitrate (NH_4_NO_3_) directly after snow melt.

### Estimation of vascular plant and moss litter production

We estimated the annual litter produced by the vegetation present in each plot during one complete year, 17 years after the start of the experiment. We assume this sampling year was representative of current litter because, a) litter production is closely linked to plant production, b) both tree and understory plant production have remained very stable in response to experimental treatments in the past 5 and 7 years across the plots, respectively [16,48,55]. In spring 2012, six litter tray traps were systematically established on the forest floor five meters from the plot center (i.e. in the N, NE, NW, S, SE and SW direction). We collected litter between the start of June 2012 and the end of May 2013. Litter traps were emptied once a month during the growing season until October, and emptied again following snow melt at the end of May. Each litter tray trap had a surface area of 0.22 m^2^ and was raised at *ca*. 2 cm above the forest floor to avoid direct contact with the soil. Small drainage holes covered in nylon mesh (1 x 1 mm) were present in the base of the litter traps in order to prevent water accumulation. These tray traps were used to collect litter fall from the following categories: *P*. *abies* needles, *P*. *sylvestris* needles, deciduous tree leaves, *V*. *myrtillus* leaves, coniferous reproductive organs (male cones, seeds) and twigs with a diameter < 0.5cm.

In addition to litter tray traps, we also systematically established six 2 x 2 m sub-plots within each plot designed to estimate the input of dead branches ≥ 0.5cm. These sub-plots were placed seven meters from the plot center (i.e. in the N, NE, NW, S, SE and SW direction), marked with stakes, and cleared of any pre-existing branches within this diameter class. This allowed us to identify and collect any new branch material that landed in these plots.

Within each plot, litter collected from different sub-plots and collection period was combined in order to provide an estimate of annual litter production for each plot. The litter was dried at 60°C for 72h, sorted into each litter category, and the mass was measured. Finally, we estimated the annual litter produced in each plot 17 years after the start of the experiment by scaling up the mass of each litter category to a surface area basis (kg or Mg ha^-1^ yr^-1^).

Because the litter tray traps were not an effective design to quantify litter production of *V*. *myrtillus* (a dwarf-shrub) or mosses, we instead quantified their litter production indirectly by estimating their annual aboveground production, which is approximately equal to their litter production since their biomasses have remained relatively stable for the last 10 years of the experiment [48]. For *V*. *myrtillus*, we estimated aboveground annual production by harvesting leaves in late June 2013 from five sub-plots sized 0.20 x 0.60 cm distributed within each treatment plot. The collected plant material was dried (60°C for 72h), and biomass from the five sub-plots were pooled, resulting in a single measurement per treatment plot. *Vaccinium myrtillus* leaf litter production per unit area (i.e. assumed to be approximately equivalent to *V*. *myrtillus* biomass production) was estimated by scaling up the total biomass from all sub-plots to a per hectare basis.

Litter production of the moss layer was estimated indirectly by evaluating vertical moss productivity [56,57]. This indirect measurement of moss tissue production could be used because moss biomass has remained stable in the different N treatments for the past 7 years [48], which means that apical moss growth is balanced by distal moss litter production [57,58]. The annual moss production was estimated by systematically selecting five intact moss patches within each plot 12 meters from the plot center, on which we established 20×20 cm quadrats (i.e. 5×0.04 m^2^ sub-plots per plot). For each of these sub-plots we positioned a plastic mesh screen (1×1cm mesh size) horizontally on the moss carpet to serve as a time zero reference. Mosses were able to freely grow through the mesh material, and in June 2014 (2 years after setting up the mesh quadrats), all moss biomass above the mesh quadrats was harvested, in order to estimate moss production. The collected moss material was dried (60°C for 72h), and biomass from the five sub-plots were pooled, resulting in a single measurement per plot. Moss litter production per unit area (i.e. assumed to be approximately equivalent to moss biomass production) was estimated by first scaling up the total biomass from all sub-plots to a per hectare basis. These values were then multiplied by the percent cover of mosses in each plot, as previously reported by [16,30]. These values were further converted to moss litter production per year by accounting for the two years over which moss biomass production was measured.

### Chemical analyses

For each litter type collected from litter traps, the dried composite samples for each plot were homogenized, and sub-samples were used for chemical characterization. For mosses, we used the newly produced biomass for chemical analysis because mosses do not exhibit discreet senescence as vascular plants do; whereas senesced tissues were used for all other litter categories. Sub-samples of each tissue type from each plot were ground using a ball mill (Retsch MM 301; Haan, Germany) and analyzed for C and N content by dry combustion using an elemental analyzer (LECO TruSpec CN analyzer; St. Joseph, MI, USA), and total P content by acid digestion using nitric-perchloric acid analyzed by inductively coupled plasmography [59]. Tissues were also analyzed for lignin, cellulose and hemi-cellulose contents by acid digestion and calcination all performed by the Soil, Water and Plant Testing Laboratory, Colorado State University, USA [60]. The C, N, P, lignin, cellulose and hemi-cellulose concentrations were expressed as mg g^-1^. For all litter categories, we estimated their annual chemical (i.e. lignin, cellulose, hemi-cellulose) and elemental (i.e. C, N and P) fluxes by multiplying the annual mass per unit area of tissue type by their corresponding chemical and elemental contents.

### Statistical analyses

All response variables were tested for normality and homoscedasticity prior to statistical analyses. Data were Box-Cox transformed when these assumptions were not met [61,62]. Variables that did not comply with these assumptions after transformation were tested using Kruskal-Wallis non-parametric test. For all other response variables which met standard parametric assumptions, we used one-way analysis of variance (ANOVAs), with N addition serving as a fixed factor and block as a random factor whenever necessary. Tree species abundance was initially used as a covariate factor but all analyses were subsequently re-run without this co-variate because it never improved the analysis. When significant differences between N addition levels were detected (α = 0.05), *post hoc* pairwise comparisons between treatments were conducted using the Student-Newman-Keuls test (for ANOVA) or Wilcoxon Ranks tests with Bonferroni correction (For K-W non-parametric test) were conducted [63]. All univariate analysis described above were performed using SPSS (Chicago, Illinois, USA; version 20.0). Data transformation was performed using XLSTAT (Addinsoft, 1995–2015; version 2015.2.02).

## Results

### Litter chemistry

The N treatments had significant effects on element stoichiometry for 2 of the 8 litter categories including mosses, and reproductive organs (Table 1); whereas, there were no differences for the carbon chemistry variables (S1 Table). Moss N concentrations significantly increased by ~18% and ~81% in the low and high N treatments relative to the control, respectively; whereas, moss P concentration decreased significantly by ~32% in the high N treatment relative to the control (Table 1). We also found that the moss C:N ratio was significantly reduced by ~14% in the low N treatment and by ~45% in the high N treatment relative to the control. In contrast, we found significant increases of both C:P and N:P ratios in the moss in the high N treatment relative to the control (Table 1).

**Table 1:** Litter stoichiometry.

	Nitrogen deposition (kg N ha^-1^yr^-1^)								
	0		12.5		50		*F*-value	DF	*P*-value
**Moss**									
%C	49.72	±0.09	49.98	±0.06	49.89	±0.11	2.32	2,12	0.141
%N	0.90**a**	±0.04	1.06**b**	±0.04	1.63**c**	±0.05	71.10	2,12	**<0.001**
%P	0.19**b**	±0.01	0.16**ab**	±0.01	0.13**a**	±0.02	5.83	2,8	**0.027**ᶲ
C:N	55.50**c**	±2.26	47.68**b**	±2.15	30.75**a**	±1.01	44.56	2,12	**<0.001**
C:P	267.11**a**	±11.77	313.74**ab**	±32.73	440.81**b**	±79.66	4.77	2,8	**0.043**ᶲ
N:P	4.85**a**	±0.30	6.68**a**	±0.85	14.60**b**	±2.91	8.70	2,12	**0.005**
***Vaccinium myrtillus* leaves**									
%C	52.12	±0.76	51.14	±1.37	51.25	±1.15	0.23	2,12	0.801
%N	1.01	±0.33	1.33	±0.39	1.29	±0.21	0.31	2,12	0.742
%P	0.09	±0.03	0.13	±0.05	0.12	±0.02	0.43	2,12	0.658
C:N	140.82	±87.76	53.71	±14.05	44.75	±7.83	1.06	2,12	0.376
C:P	1347.79	±786.23	695.61	±221.85	470.94	±83.56	0.92	2,12	0.424
N:P	10.92	±1.25	11.99	±1.35	10.50	±0.29	0.51	2,12	0.612
**Reproductive organs**									
%C	54.10**b**	±0.39	51.20**a**	±0.99	51.47**a**	±1.20	4.56	2,12	**0.048**ᶲ
%N	1.30**b**	±0.31	0.60**a**	±0.23	0.66**a**	±0.09	6.64	2,12	**0.020**ᶲ
%P	0.13**b**	±0.03	0.06**a**	±0.02	0.06**a**	±0.01	4.42	2,12	**0.019**ᶲ
C:N	54.84**a**	±15.77	139.89**b**	±39.03	86.46**ab**	±15.04	4.52	2,8	**0.049**ᶲ‡
C:P	560.76	±176.25	1571.75	±462.76	1025.18	±222.77	2.61	2,12	0.115
N:P	10.22	±0.81	10.97	±0.62	11.56	±0.59	0.99	2,12	0.401
**Twig litter**									
%C	52.44	±0.49	52.27	±1.56	51.63	±1.51	0.11	2,12	0.895
%N	1.23	±0.33	0.81	±0.21	0.97	±0.27	0.62	2,12	0.557
%P	0.10	±0.03	0.07	±0.02	0.10	±0.03	0.25	2,12	0.781
C:N	58.63	±16.52	85.18	±20.38	90.52	±39.70	1.28	2,12	0.527
C:P	799.10	±214.05	981.73	±278.29	1029.89	±512.02	0.12	2,12	0.892
N:P	15.03	±4.11	11.19	±0.77	10.35	±0.70	1.03	2,12	0.385
**Branch litter**									
%C	51.25	±1.10	51.03	±1.19	51.47	±1.13	0.04	2,12	0.965
%N	1.43	±0.38	1.44	±0.28	0.85	±0.40	0.73	2,12	0.506
%P	0.10	±0.04	0.15	±0.03	0.05	±0.03	2.19	2,12	0.154
C:N	46.49	±10.44	42.81	±10.17	92.07	±33.21	2.40	2,12	0.141
C:P	944.06	±435.82	422.82	±97.07	2426.49	±932.72	2.87	2,12	0.085
N:P	31.39	±21.35	9.90	±0.28	10.40	±0.49	0.19	2,12	0.400
**Deciduous tree leaves**									
%C	50.95	±1.02	50.97	±1.72	52.08	±1.38	0.21	2,12	0.814
%N	0.79	±0.34	0.92	±0.23	0.87	±0.23	0.06	2,12	0.946
%P	0.07	±0.03	0.09	±0.03	0.08	±0.03	0.16	2,12	0.854
C:N	139.79	±44.49	68.18	±13.13	77.87	±19.41	1.79	2,12	0.209
C:P	1638.83	±573.06	792.02	±192.48	1023.31	±321.65	1.23	2,12	0.328
N:P	11.75	±0.98	11.13	±0.84	12.41	±1.20	0.39	2,12	0.684
***Picea abies* needles**									
%C	51.42	±1.02	51.57	±1.36	51.53	±1.18	0.00	2,12	0.996
%N	1.12	±0.26	1.71	±0.35	1.76	±0.34	0.96	2,12	0.410
%P	0.10	±0.01	0.17	±0.03	0.12	±0.04	1.47	2,12	0.268
C:N	55.18	±10.04	36.55	±7.91	76.33	±33.49	2.58	2,12	0.275†
C:P	565.42	±67.01	361.69	±72.13	794.69	±359.29	1.01	2,12	0.392
N:P	10.94	±0.99	10.10	±0.38	10.15	±0.29	0.56	2,12	0.588
***Pinus sylvestris* needles**									
%C	52.27	±1.07	50.30	±1.16	51.10	±1.03	0.83	2,12	0.460
%N	0.80	±0.32	1.14	±0.38	1.01	±0.34	0.25	2,12	0.779
%P	0.08	±0.03	0.11	±0.04	0.10	±0.04	0.18	2,12	0.838
C:N	109.02	±33.07	155.02	±112.71	93.08	±42.98	0.96	2,12	0.619†
C:P	1084.41	±281.47	1589.86	±1138.07	1310.52	±752.25	0.10	2,12	0.906
N:P	10.28	±0.74	10.51	±0.22	11.81	±2.78	0.96	2,12	0.410

Mean (±SE) concentrations of carbon, nitrogen and phosphorus (mg g^-1^) and associated ratios for 8 litter categories in response to chronic nitrogen deposition (0, 12.5, and 50 kg N ha^-1^ yr^-1^). Each variable was estimated in replicated (n = 5) 0.1 ha plots 17 years after treatments were initiated. The F- and P-values were derived from a one-way ANOVA for each variable, and the letters a, b or c indicate significant differences determined by using Student-Newman-Keuls *post-hoc* analyses.

† Kruskal-Wallis non-parametric test was used, and in case of significance pairwise *post-hoc* Wilcoxon Ranks test was conducted using the letters a or b.

‡ Statistical analyses were performed on box-cox transformed data.

ᶲ Block was used as a significant factor.

Values in bold indicate statistical significances at P < 0.05.

For coniferous reproductive organs (male cones and seeds), we found a significant decrease in %C, %N and %P for the two N treatments relative to the control, and a significant increase in the C:N ratio of the low N treatment relative to the control. No significant differences were found for twig tissues, except a nearly significant increase in the lignin:N ratio for the low and high N treatments relative to the control (S1 Table). For tree branch litter, we found a nearly significant increase in the C:P ratio in the high N treatment relative to the control. Finally, *P*. *sylvestris* needle tissues showed marginal difference for the lignin:N ratio towards a decrease in the high N treatment relative to the control (S1 Table). These decreases were mainly due to a non-significant downward trend in the %lignin of *P*. *sylvestris* needle tissues.

### Litter quantities

**Fig 1:**
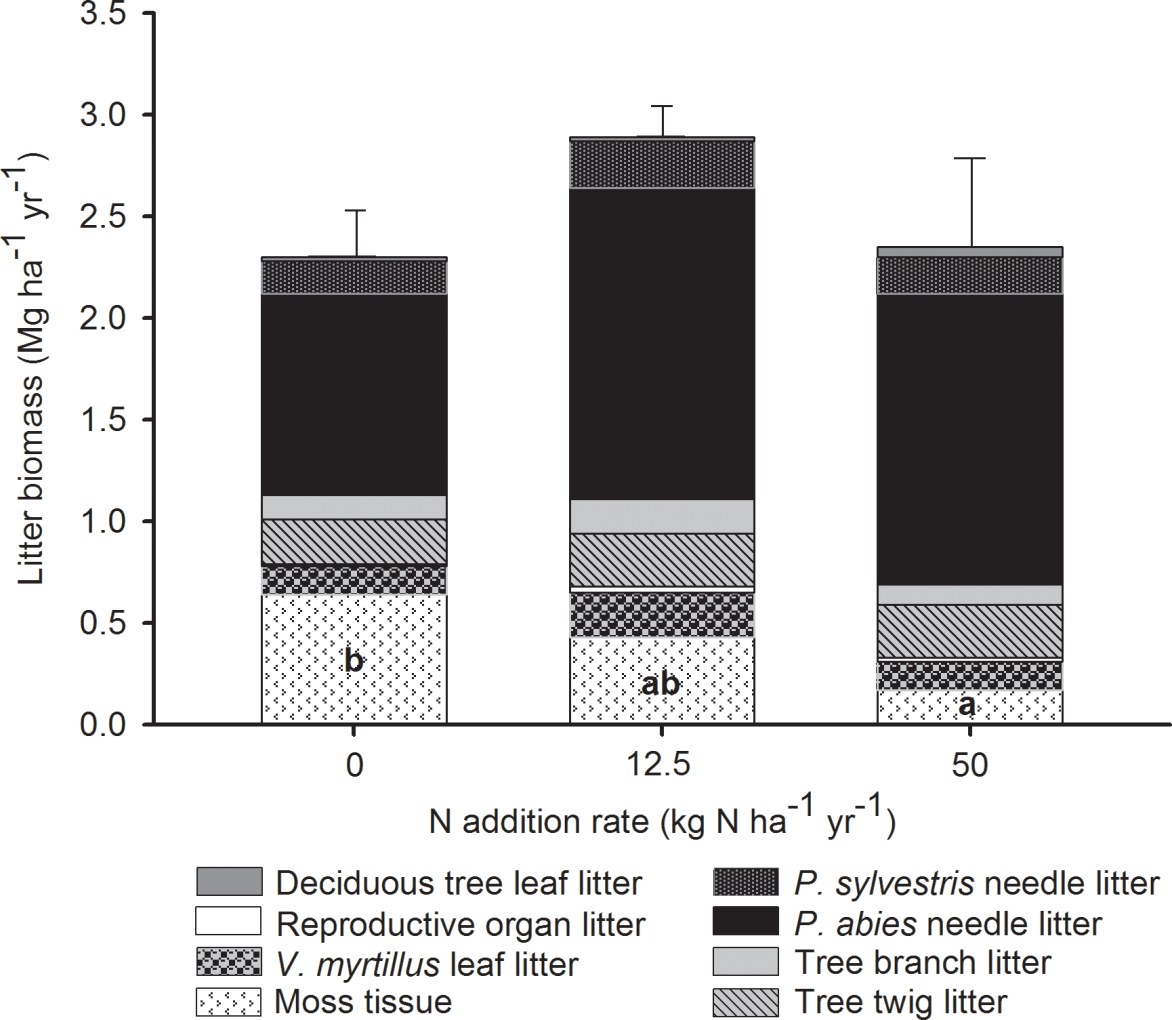
Mean annual litter biomass in response to nitrogen addition. Mean (Mg ha^-1^ yr^-1^) biomass of 8 litter categories (moss tissue, *V*. *myrtillus* leaf, reproductive organ, tree twig, tree branch litter, *P*. *abies* needle, *P*. *sylvestris* needle and deciduous tree leaf litter) in response to long-term N addition (0, 12.5 or 50 kg N ha-1 yr-1; n = 5). The total bar height represents the total (+SE) litter biomass (Mg ha^-1^ yr^-1^). Different letters (a or b) across bar segments with the same shade indicate significant differences pair-wise difference using Student-Newman-Keuls *post-hoc* tests.

Our data did not reveal any significant differences in litter quantities, except for moss litter, which was non-significantly reduced by ~33% (0.21 Mg ha^-1^ yr^-1^) in the low N treatment, and significantly reduced by ~73% (0.47 Mg ha^-1^ yr^-1^) in the high N treatment relative to the control (Fig 1, S2 Table). The total annual litter biomass ranged between 2.29 and 2.86 Mg ha^-1^ among all plots, where *P*. *abies* and *P*. *sylvestris* needles in combination accounted for ~60% of the total litter produced annually (Fig 1, S2 Table). For this needle litter, we found that the N treatments caused a nearly significant increase of ~53% (0.61 Mg ha^-1^) in the low N treatment and ~40% (0.46 Mg ha^-1^) in the high N treatment, both relative to the control (F = 3.38, P = 0.068).

### Element and C chemistry fluxes

**Fig 2:**
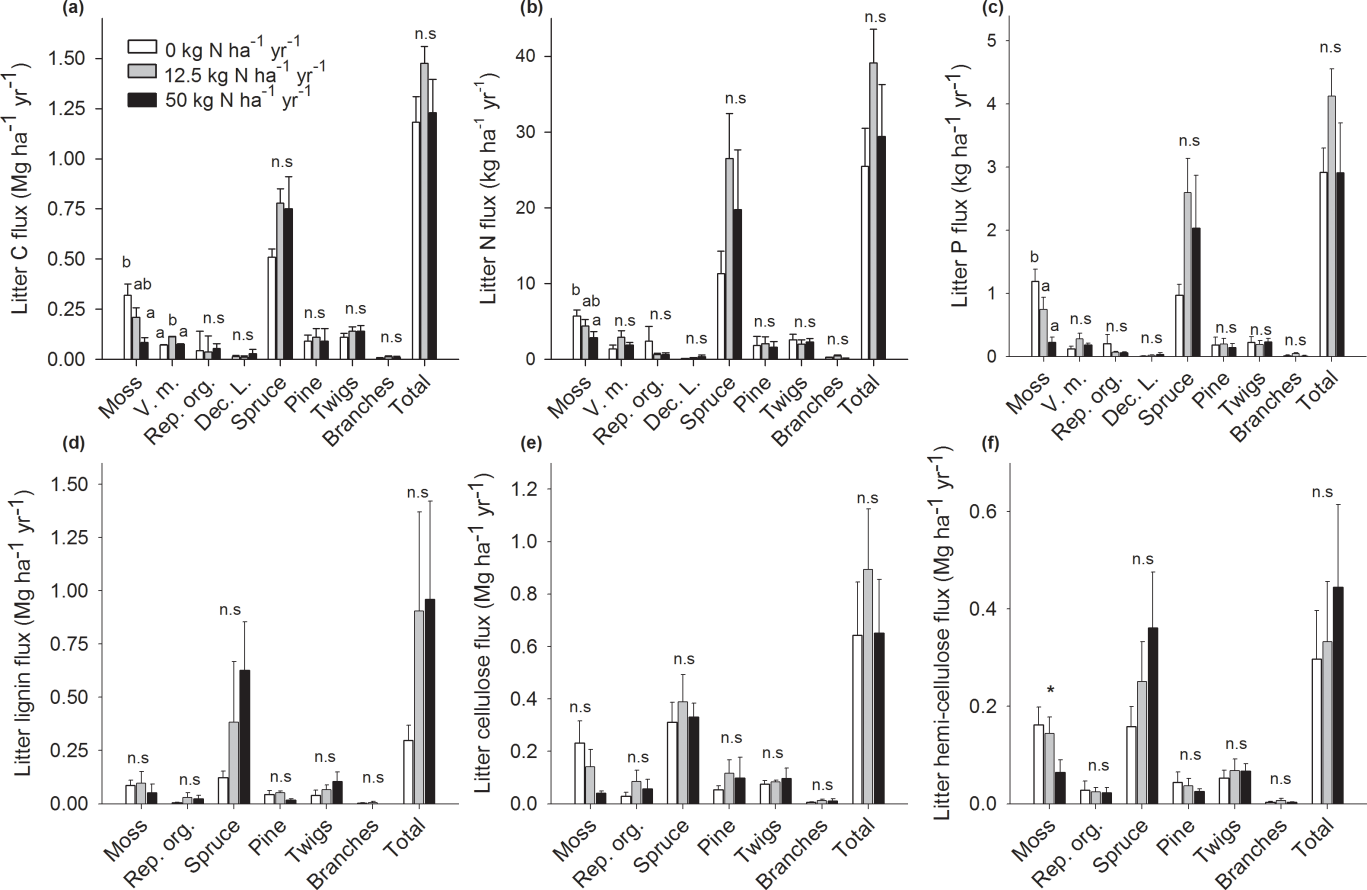
Litter element and carbon chemistry fluxes. Total mean (+SE) litter carbon (a), nitrogen (b), phosphorus (c), lignin (d), cellulose (e) or hemi-cellulose (f) fluxes in response to long-term N addition (0, 12.5 or 50 kg N ha^-1^ yr^-1^; n = 5) for each litter category: moss, *V*. *myrtillus* leaves (V. m.), reproductive organs (Rep. org.), deciduous tree leaves (Dec. L.), *P*. *abies* needles (Spruce), *P*. *sylvestris* (Pine), twigs and branches. V. m and Dec. L. litter categories are missing from panels (d), (e) and (f) because insufficient litter material was available for these analyses. Different letters (a or b) next to each group of bars indicate significant differences between treatments (α = 0.05) determined using Student-Newman-Keuls *post-hoc* tests. Nearly significant difference at (0.05 < P < 0.10) are indicated by a star (*). Non-significant differences are indicated by n.s.

In addition to total annual litter production, estimates of annual litter fluxes of C, N, P, lignin, cellulose and hemi-cellulose were also not significantly different among treatments for most litter categories, except for the moss and *V*. *myrtillus* leaves litter (Fig 2, S2 Table). We found that the moss C flux non-significantly decreased by ~34% (0.11 Mg ha^-1^ yr^-1^) in the lower N treatment and significantly decreased by ~74% (0.24 Mg ha^-1^ yr^-1^) in the high N treatment relative to the control (Fig 2A, S2 Table). For the moss N flux, we found a significant decrease in the high N treatment relative to the control. Concerning the moss P flux, we found significant decreases by ~81% (0.96 kg ha^-1^ yr^-1^) in the high N treatment relative to the control (Fig 2B, S2 Table). We also found a marginal downward trend of the moss hemi-cellulose flux towards a decrease in the high N treatment relative to the control. Finally, the C flux in *V*. *myrtillus* leaf litter significantly increased by ~36% (0.40 Mg ha^-1^ yr^-1^) in the low N treatment relative to the control, while the high N treatment showed no differences with the control (Fig 2A, S2 Table).

We found few differences in total fluxes when individual litter categories of C, N, P, lignin, cellulose, and hemi-cellulose fluxes were summed into understory, overstory, and total fluxes, except for the understory C, N and P fluxes (Table 2). We found that the high N treatment caused a significant decrease by ~59% (0.23 Mg ha^-1^ yr^-1^) for the understory C flux. We also found the high N treatment caused a significant decrease by ~33% (2.37 kg ha^-1^ yr^-1^) for the understory N flux. Finally, the understory P flux showed a significant decrease in the high N treatment relative to the control and the low N treatment (Table 2).

**Table 2:** Total litter fluxes.

	Nitrogen deposition (kg N ha^-1^yr^-1^)								
	0		12.5		50		*F*-value	DF	*P*-value
**Understory fluxes**									
Understory C flux (Mg ha^-1^ yr^-1^)	0.39**b**	±0.05	0.32**b**	±0.05	0.16**a**	±0.02	7.78	2,12	**0.007**
Understory N flux (kg ha^-1^ yr^-1^)	7.09**b**	±0.61	7.30**b**	±1.29	4.72**a**	±0.57	5.50	2,8	**0.031**ᶲ
Understory P flux (kg ha^-1^ yr^-1^)	1.32**b**	±0.18	1.02**b**	±0.21	0.41**a**	±0.07	7.95	2,12	**0.006**
**Overstory fluxes**									
Tree C flux (Mg ha^-1^ yr^-1^)	0.79	±0.09	1.15	±0.04	1.07	±0.17	2.73	2,12	0.105
Tree N flux (kg ha^-1^ yr^-1^)	18.36	±4.89	31.81	±4.96	25.92	±9.81	1.47	2,12	0.277
Tree P flux (kg ha^-1^ yr^-1^)	1.60	±0.35	3.10	±0.45	2.50	±0.74	1.94	2,12	0.187
Tree lignin flux (Mg ha^-1^ yr^-1^)	0.20	±0.07	0.76	±0.47	0.90	±0.24	1.52	2,9	0.283
Tree cellulose flux (Mg ha^-1^ yr^-1^)	0.42	±0.10	0.72	±0.16	0.61	±0.21	0.83	2,11	0.467
Tree hemi-cellulose flux (Mg ha^-1^ yr^-1^)	0.30	±0.09	0.33	±0.12	0.45	±0.17	0.33	2,11	0.728
**Total fluxes**									
Total C flux (Mg ha^-1^ yr^-1^)	1.18	±0.13	1.48	±0.08	1.23	±0.16	1.47	2,12	0.267
Total N flux (kg ha^-1^ yr^-1^)	25.46	±5.05	39.12	±4.49	29.39	±6.86	1.60	2,12	0.242
Total P flux (kg ha^-1^ yr^-1^)	2.92	±0.38	4.12	±0.43	2.91	±0.78	1.53	2,12	0.255
Total lignin flux (Mg ha^-1^ yr^-1^)	0.30	±0.07	0.90	±0.46	0.96	±0.22	1.57	2,9	0.274
Total cellulose flux (Mg ha^-1^ yr^-1^)	0.64	±0.20	0.89	±0.23	0.65	±0.20	0.44	2,11	0.656
Total hemi-cellulose flux (Mg ha^-1^ yr^-1^)	0.30	±0.09	0.33	±0.12	0.44	±0.17	0.33	2,11	0.728

Mean (±SE) of carbon (C), nitrogen (N), phosphorus (P), lignin, cellulose and hemi-cellulose total fluxes for understory, overstory, and total fluxes in response to chronic nitrogen deposition (0, 12.5, and 50 kg N ha^-1^ yr^-1^). Each variable was estimated in replicated (n = 5) 0.1 ha plots 17 years after treatments were initiated. The F- and P-values were derived from a one-way ANOVA for each variable, and the letters a or b indicate significant differences determined using Student-Newman-Keuls *post-hoc* analyses.

ᶲ block was used as a significant factor.

Values in bold indicate statistical significances at P < 0.05.

## Discussion

Our data derived from a unique long-term experiment indicate only minor effects of chronic N addition on the contemporary quantity and quality of annual litter produced by boreal forest vegetation, even at a relatively high N addition rate (50 kg N ha^-1^ yr^-1^). We show that understory litter C, N and P fluxes were the most responsive to chronic N addition, whereas litter production of multiple categories of canopy litter was largely unresponsive to N addition.

Inconsistent with our first hypothesis, our data showed that long-term N addition did not increase the litter N concentration for a majority of the litter types we measured, except for moss tissue. The unresponsiveness of litter N concentrations to treatments sharply contrast previous measurements of green leaves in our experimental system (e.g. [15,42]), where significant increases of N concentrations of *P*. *abies* and *V*. *myrtillus* were found in the high N treatment relative to the control. The absence of elevated N concentrations in the litter of these vascular plant species indicates that they effectively resorb N prior to litter senescence [33,64]. This suggests that plants remain N limited at our experimental site, even when subjected to the high N treatment for 17 years. A previous study in the same system showed that approximately 50% of the total N added for each N treatment after 16 years remains in the organic soil horizon [18]. Persistent N limitation despite a large increase in soil N suggests a majority of the N added to the soil is largely unavailable for plant uptake, which could occur as a result of abiotic (e.g. reaction between N and phenolics) or biotic soil retention [65].

Also contrary to our first hypothesis, coniferous reproductive structures showed a decreases in N concentration in response to N addition as well as a significant increase of the C:N ratios in response to N addition. While it is generally expected that N addition should increase the decomposability of plant litter due to increase in litter N concentrations [32], our data suggest that the stoichiometry of many types of plant litter is not always responsive, and sometimes even becomes less N enriched. These findings highlight that litter may not be as impacted by N deposition as it is sometimes suggested to be [33], which subsequently may have little effect on decomposition.

Consistent with our first hypothesis, we found that the N concentration of moss tissues increased, which is indicative of their ability to intercept a substantial portion of N deposition. Because mosses produce very recalcitrant litter, they are able to convert this reactive inorganic N into a stable soil organic N pool, and thus also limit its uptake by vascular plants [9,50,66]. Further, bryophytes were the only tissue type evaluated to show a decreased C:N ratio in response to the low and high N treatments, which is potentially important because bryophytes are known to have very slow decomposition rates relative to vascular plant litter [66,67], and thus are considered as important contributors to soil C accumulation [9,68]. Further, bryophytes have been reported to have very low N losses due to their efficiently to recycle nutrients in their tissues, with N residence times in mosses estimated to range from 3 to 10 years [68].While N resorption during moss tissue senescence reduces these ratios somewhat [68,69], lower C:N ratios of moss litter as a result of atmospheric N deposition could potentially diminish their recalcitrance [66,67], and thus reduce their contribution to soil C accumulation [9].

Inconsistent with our first hypothesis, we found a decrease of P concentration in reproductive structures. This result contrasts with other studies that have shown increased plant P concentrations in response to N addition, which has been proposed to occur as a result of increased root and microbial phosphatase activities, that allow plants to achieve a more balanced N:P stoichiometry when N is in surplus [70,71]. Further, we found a decrease of P concentration and an increase in C:P ratios in moss tissues. This could be indicative of their inefficiency to take up P. Feather mosses gain P mainly through precipitation and canopy throughfall rather than from the soil [68,72]. This finding is consistent with Phuyal et al. [73], where they did not find any change in moss tissue P assimilation in response to N addition, despite higher phosphatase activity in their tissues.

Regarding our second hypothesis, we found only marginally significant evidence that annual tree litter (coniferous or deciduous) production increased after 17 years of N addition. The two most abundant sources of aboveground litter, *Picea* and *Pinus* needles, did not show any change with N addition when considered individually; however, when considered together they showed a nearly significant increase in response to both low and high N addition rates. This result is consistent with Frey et al.[17], where high N addition rates (50–150 kg N ha^-1^ yr^-1^) caused an increase of needle litter by 18% in a long-term northern pine forest experiment (20 years), which the authors attributed to higher tree mortality as well as a reduction of needle life span [25]. This result is also consistent with Krause et al. [26], who showed a relatively low N addition rate (23 kg N ha^-1^ yr^-1^) caused an increase of needle biomass and area in a long-term alpine spruce forest experiment (14 years).

In support of our second hypothesis, our data showed that 17 years after N addition, moss litter production decreased by ~73%, but only in response to the high N treatment (50 kg N ha^-1^ yr^-1^). We did not find a similar decrease for the other major fraction of understory litter that derived from *V*. *myrtillus*. Several other N fertilization studies have observed decreases in moss biomass [50,74–76], which have coincided with increases in moss tissue N concentrations, as shown in our experimental system [18,30]. This decline in moss biomass has been attributed to a direct toxicity of NH_4_^+^ on bryophyte tissues [74,75] and light competition with vascular plants [76].

Contrary to our third hypothesis, our data showed that 17 years of N addition did not increase the total aboveground litter fluxes of C, N, P, or C chemical forms (i.e. lignin, hemi-cellulose, or cellulose). Instead, we found that the nearly significant increase in conifer needle production was diluted by the unresponsiveness of other overstory litter categories; and further, was offset by significantly lower understory litter fluxes in the N treatments. The significant decrease in the total understory C, N and P litter fluxes occurred because mosses were significantly less abundant in N treated plots compared to the control plots, where they contributed between 53–82% of the understory litter annually produced at the experimental site. The decline in moss litter production had an over-riding effect on *V*. *myrtillus*, which increased by ~36% in the low N treatment relative to the control. These data highlight that the element fluxes of some specific litter categories can be highly responsive to N addition, whereas the total fluxes may be largely unresponsive due to off-setting responses of some litter categories.

## Conclusions

Our results have substantial implications for better understanding the mechanisms through which atmospheric N deposition can influence soil C sequestration in boreal regions. We show that litter chemistry was generally not affected by N addition, suggesting a continued presence of N limitation to plant growth even after 17 years of substantial N addition. A recent meta-analysis has shown that anthropogenic N addition sometimes has positive or negative effect on plant litter inputs (average effect was neutral) [21]. However, a majority of the data underpinning this meta-analysis were derived from experiments using much higher N doses than the upper N deposition limit in the regions of study, leaving some uncertainty regarding the impacts of more realistic chronic N deposition rates. Our result is the longest running experiment simulating realistic N_r_ deposition levels in the boreal region (12.5 kg N ha^-1^ yr^-1^), and show a minimal impact on contemporary aboveground litter C inputs; whereas, higher N addition rates (50 kg N ha^-1^ yr^-1^) caused understory litter C inputs to decrease, especially for bryophytes.

Previous work from our study system has shown that the long-term N addition treatments have caused soil C to increase at a rate of 10 kg C kg^-1^ N added [18]. Thus, a 30% increase in litter production (0.69 Mg ha^-1^ yr^-1^) would be required to explain the observe increase of the soil C pool. Therefore, our current study provided little evidence that enhanced contemporary litter production was unlikely to explain this observed increase in C, and suggests other mechanisms are responsible. Our measurements of litter inputs are representative of current vegetation production in our plots, which have remained stable in response to the N treatment for 5 or 7 years for trees and understory, respectively. However, higher tree production in response to the high N treatment was observed during the first 10 years of the experiment, which may have corresponded with higher litter production rates during that period that may have contributed to the increased soil C observed in these plots today. An additional mechanism that likely explains enhanced soil C in both the high and low N plots is a change in microbial mediated soil processes. For example, N enrichment may have changed the activity and abundance of soil biota [18,77–79], resulting in slower decomposition and reduced soil respiration rates [18,80–82], and thus higher rates of soil C accumulation. Further, belowground litter inputs (e.g. exudates, fine roots, fungal tissues) may be important for understanding soil C accumulation rates, which we did not consider in the current study[21]. Complementary work on these additional mechanisms are needed in order to provide a more complete understanding of how net ecosystem C exchange responds to atmospheric N deposition.

## Supporting Information

S1 Deposited Data (ZIP)

S1 Table Mean (±SE) concentrations of lignin, cellulose and hemi-cellulose (mg g^-1^) and associated ratios for 8 litter categories in response to chronic nitrogen deposition (0, 12.5, and 50 kg N ha^-1^ yr^-1^). Each variable was estimated in replicated (n = 5) 0.1 ha plots 17 years after treatments were initiated. The F- and P-values were derived from a one-way ANOVA for each variable, and the letters a or b indicate significant differences determined using Student-Newman-Keuls *post-hoc* analyses.
(DOCX)

S2 Table The F-values, degrees of freedom (df), and P-values from one-way ANOVAS comparing litter biomasses and litter fluxes across three nitrogen deposition treatments (0,12 .5, and 50 kg N ha^-1^ yr^-1^). (DOCX)
