# Supplementary material for: Chronic Nitrogen Deposition Has a Minor Effect on the Quantity and Quality of Aboveground Litter in a Boreal Forest
Source: PLoS One. 2016 Aug 31;11(8):e0162086. doi: 10.1371/journal.pone.0162086 (PMC5007034; doi:10.1371/journal.pone.0162086)
Supplement: S1 Table — Each variable was estimated in replicated (n = 5) 0.1 ha plots 17 years after treatments were initiated. The F- and P-values were derived from a one-way ANOVA for each variable, and the letters a or b indicate significant differences determined using Student-Newman-Keuls post-hoc analyses. (DOCX) [file pone.0162086.s002.docx]

|  | Nitrogen deposition (kg N ha^-1^yr^-1^) | | | | | |  | | |
| --- | --- | --- | --- | --- | --- | --- | --- | --- | --- |
| Litter parameters | 0 | | 12.5 | | 50 | | *F*-value | DF | *P*-value |
| **Moss** |  |  |  |  |  |  |  |  |  |
| %Lignin | 14.85 | ±5.66 | 18.32 | ±6.99 | 23.90 | ±15.32 | 0.20 | 2,12 | 0.823 |
| %Cellulose | 32.62 | ±7.62 | 28.06 | ±6.76 | 27.48 | ±5.26 | 0.18 | 2,12 | 0.836 |
| %Hemi-cellulose | 30.88 | ±9.62 | 38.95 | ±8.43 | 32.60 | ±7.74 | 0.24 | 2,12 | 0.788 |
| Lignin:N | 16.53 | ±6.10 | 18.40 | ±7.91 | 14.52 | ±9.30 | 0.06 | 2,12 | 0.941 |
| Lignin:P | 77.26 | ±26.93 | 110.56 | ±37.65 | 216.04 | ±133.90 | 0.78 | 2,12 | 0.478 |
| Lignin:Cellulose | 0.58 | ±0.27 | 0.60 | ±0.15 | 0.97 | ±0.63 | 0.29 | 2,12 | 0.755 |
| **Reproductive organs** |  |  |  |  |  |  |  |  |  |
| %Lignin | 9.41 | ±2.87 | 35.77 | ±19.33 | 24.76 | ±10.55 | 1.07 | 2,12 | 0.375 |
| %Cellulose | 33.74 | ±7.34 | 36.07 | ±8.29 | 33.93 | ±9.83 | 0.87 | 2,11 | 0.649† |
| %Hemi-cellulose | 23.62 | ±6.15 | 18.90 | ±7.07 | 23.10 | ±8.27 | 0.39 | 2,11 | 0.825† |
| Lignin:N | 12.48 | ±7.12 | 147.00 | ±96.27 | 50.38 | ±30.27 | 3.75 | 2,11 | 0.154† |
| Lignin:P | 129.50 | ±78.55 | 654.49 | ±391.82 | 642.30 | ±408.33 | 1.05 | 2,11 | 0.387 |
| Lignin:Cellulose | 0.28 | ±0.06 | 0.83 | ±0.33 | 0.97 | ±0.44 | 2.02 | 2,11 | 0.364† |
| **Twig litter** |  |  |  |  |  |  |  |  |  |
| %Lignin | 16.34 | ±6.67 | 38.62 | ±17.54 | 38.33 | ±15.64 | 2.75 | 2,12 | 0.253† |
| %Cellulose | 37.63 | ±8.45 | 36.97 | ±8.55 | 32.13 | ±7.22 | 0.38 | 2,12 | 0.827† |
| %Hemi-cellulose | 22.17 | ±6.87 | 22.65 | ±7.01 | 27.33 | ±6.12 | 0.18 | 2,12 | 0.836 |
| Lignin:N | 17.09 | ±6.91 | 43.11 | ±9.40 | 46.77 | ±13.59 | 4.88 | 2,12 | 0.087† |
| Lignin:P | 223.46 | ±70.83 | 499.27 | ±118.95 | 497.21 | ±156.82 | 1.73 | 2,12 | 0.219 |
| Lignin:Cellulose | 0.59 | ±0.32 | 0.99 | ±0.29 | 1.35 | ±0.65 | 2.56 | 2,12 | 0.278† |
| **Branch litter** |  |  |  |  |  |  |  |  |  |
| %Lignin | 18.82 | ±7.77 | 20.80 | ±6.85 | 22.78 | ±12.24 | 0.05 | 2,12 | 0.955 |
| %Cellulose | 40.07 | ±9.91 | 44.04 | ±10.71 | 34.81 | ±8.32 | 0.23 | 2,12 | 0.799 |
| %Hemi-cellulose | 25.35 | ±9.71 | 24.73 | ±10.12 | 27.06 | ±7.63 | 0.02 | 2,12 | 0.983 |
| Lignin:N | 18.41 | ±8.63 | 17.09 | ±6.18 | 18.86 | ±10.82 | 1.01 | 2,12 | 0.988 |
| Lignin:P | 632.69 | ±377.69 | 168.29 | ±62.99 | 887.52 | ±592.03 | 0.83 | 2,12 | 0.463 |
| Lignin:Cellulose | 0.64 | ±0.41 | 0.63 | ±0.28 | 0.91 | ±0.50 | 0.15 | 2,12 | 0.860 |
| ***Picea abies* needles** |  |  |  |  |  |  |  |  |  |
| %Lignin | 13.05 | ±3.86 | 22.09 | ±15.61 | 43.37 | ±11.31 | 2.37 | 2,8 | 0.156ᶲ |
| %Cellulose | 30.07 | ±5.52 | 24.85 | ±5.39 | 24.47 | ±3.76 | 1.64 | 2,12 | 0.441 |
| %Hemi-cellulose | 17.38 | ±5.13 | 18.10 | ±6.11 | 23.28 | ±4.44 | 1.52 | 2,12 | 0.468† |
| Lignin:N | 14.47 | ±6.25 | 22.38 | ±19.15 | 71.46 | ±42.58 | 4.56 | 2,12 | 0.102† |
| Lignin:P | 143.38 | ±51.83 | 209.11 | ±175.34 | 750.37 | ±454.01 | 1.39 | 2,12 | 0.287 |
| Lignin:Cellulose | 0.55 | ±0.22 | 1.13 | ±0.85 | 1.96 | ±0.56 | 3.86 | 2,12 | 0.145† |
| ***Pinus sylvestris* needles** |  |  |  |  |  |  |  |  |  |
| %Lignin | 19.81 | ±3.65 | 19.19 | ±1.86 | 12.38 | ±3.03 | 1.97 | 2,12 | 0.196 |
| %Cellulose | 29.75 | ±8.12 | 37.27 | ±7.99 | 30.19 | ±7.45 | 0.29 | 2,12 | 0.756 |
| %Hemi-cellulose | 22.54 | ±7.78 | 15.67 | ±7.37 | 22.88 | ±7.54 | 0.29 | 2,12 | 0.756 |
| Lignin:N | 31.15 | ±6.44 | 66.13 | ±47.62 | 10.55 | ±1.52 | 5.12 | 2,9 | 0.077† |
| Lignin:P | 328.84 | ±72.37 | 679.09 | ±480.19 | 110.71 | ±12.61 | 1.05 | 2,9 | 0.391 |
| Lignin:Cellulose | 0.85 | ±0.24 | 0.60 | ±0.14 | 0.52 | ±0.17 | 1.85 | 2,9 | 0.397† |

† Kruskal-Wallis non parametric test was used, and in case of significance pairwise *post-hoc* Wilcoxon Ranks was conducted using the letters a or b. ᶲ block was used as a significant factor. Values in bold indicate statistical significances at P < 0.05.
