# Supplementary material for: Chronic Nitrogen Deposition Has a Minor Effect on the Quantity and Quality of Aboveground Litter in a Boreal Forest
Source: PLoS One. 2016 Aug 31;11(8):e0162086. doi: 10.1371/journal.pone.0162086 (PMC5007034; doi:10.1371/journal.pone.0162086)
Supplement: S2 Table — (DOCX) [file pone.0162086.s003.docx]

|  | *F*-value | DF | *P*-value |
| --- | --- | --- | --- |
| Moss litter biomass | 7.17 | 2,12 | **0.009** |
| C flux | 7.33 | 2,12 | **0.008** |
| N flux | 5.54 | 2,8 | **0.031**ᶲ |
| P flux | 8.93 | 2,12 | **0.004** |
| Lignin flux | 0.29 | 2,12 | 0.749 |
| Cellulose flux | 2,40 | 2,12 | 0.132 |
| Hemi-cellulose flux | 3.63 | 2,8 | 0.076ᶲ |
| *Vaccinium myrtillus* leaves biomass | 0.94 | 2,15 | 0.415 |
| C flux | 112.22 | 2,12 | **<0.001** |
| N flux | 1.74 | 2,12 | 0.217 |
| P flux | 1.59 | 2,12 | 0.244 |
| Reproductive organ biomass | 0.31 | 2,12 | 0.740 |
| C flux | 0.27 | 2,12 | 0.766 |
| N flux | 0.76 | 2,12 | 0.491 |
| P flux | 0.76 | 2,12 | 0.490 |
| Lignin flux | 0.92 | 2,10 | 0.431 |
| Cellulose flux | 0.74 | 2,11 | 0.499 |
| Hemi-cellulose flux | 0.03 | 2,11 | 0.966 |
| Twig litter biomass | 0.38 | 2,12 | 0.694 |
| C flux | 0.31 | 2,12 | 0.742 |
| N flux | 0.21 | 2,12 | 0.811 |
| P flux | 0.10 | 2,12 | 0.903 |
| Lignin flux | 0.99 | 2,11 | 0.404 |
| Cellulose flux | 0.19 | 2,12 | 0.834 |
| Hemi-cellulose flux | 0.21 | 2,12 | 0.814 |
| Branch litter biomass | 0.87 | 2,12 | 0.443 |
| C flux | 0.71 | 2,12 | 0.510 |
| N flux | 1.27 | 2,12 | 0.324 |
| P flux | 2.21 | 2,12 | 0.153 |
| Lignin flux | 1.35 | 2,11 | 0.299 |
| Cellulose flux | 0.44 | 2,12 | 0.657 |
| Hemi-cellulose flux | 0.72 | 2,12 | 0.507 |
| Deciduous tree leaf biomass | 0.95 | 2,12 | 0.427ᶲ |
| C flux | 0.54 | 2,12 | 0.595 |
| N flux | 1.15 | 2,12 | 0.350 |
| P flux | 0.96 | 2,12 | 0.412 |
| *Picea abies* needle biomass | 2.19 | 2,12 | 0.155 |
| C flux | 2.06 | 2,12 | 0.171 |
| N flux | 1.65 | 2,12 | 0.233 |
| P flux | 1.82 | 2,12 | 0.204 |
| Lignin flux | 1.44 | 2,12 | 0.275 |
| Cellulose flux | 0.25 | 2,12 | 0.780 |
| Hemi-cellulose flux | 1.43 | 2,12 | 0.278 |
| *Pinus sylvestris* needle biomass | 1.37 | 2,12 | 0.856 |
| C flux | 0.09 | 2,12 | 0.912 |
| N flux | 0.05 | 2,12 | 0.949 |
| P flux | 0.09 | 2,12 | 0.914 |
| Lignin flux | 1.96 | 2,9 | 0.197 |
| Cellulose flux | 0.33 | 2,9 | 0.730 |
| Hemi-cellulose flux | 0.34 | 2,9 | 0.720 |

ᶲ block was used as a significant factor. Values in bold indicate statistical significances at P < 0.05.
